# Diagnostic Pitfall of Lytic Bone Lesions: A Case of Chromophobe Renal Cell Carcinoma Coexisting With Multiple Myeloma

**DOI:** 10.7759/cureus.95985

**Published:** 2025-11-03

**Authors:** Aaeshah M Almutairi, Omar Abusedera, Adnan M Shaheen

**Affiliations:** 1 Medical School, Royal College of Surgeons in Ireland - Bahrain, Muharraq, BHR; 2 Urology, Royal College of Surgeons in Ireland - Bahrain, Bahrain Defence Force Hospital, Muharraq, BHR

**Keywords:** back pain, chromophobe renal cell carcinoma, lytic bone lesions, misdiagnosis, multiple myeloma

## Abstract

The coexistence of renal cell carcinoma (RCC) and multiple myeloma (MM) is rare and presents a significant diagnostic challenge, particularly when patients present with back pain and lytic bone lesions. Several case series and reports have documented a reciprocal relationship between the two malignancies, which may be attributed to shared etiological factors, analogous cytokine-mediated growth mechanisms, and converging clinical presentations. We report the case of a 66-year-old man diagnosed with chromophobe RCC who presented with persistent back pain and was ultimately diagnosed with coexisting MM.

## Introduction

Chromophobe renal cell carcinoma (ChRCC), a rare subtype accounting for approximately 5% of all renal cell carcinoma (RCC) cases, typically presents in the sixth decade of life and is associated with favorable long-term outcomes due to its indolent behavior and low metastatic potential [1]. Incidence of ChRCC is similar in both men and women, and most cases are diagnosed at an early stage, often incidentally during imaging for unrelated reasons [2]. Clinical symptoms such as hematuria, flank pain, and palpable mass are infrequent and usually occur only in advanced disease. When metastases do occur, the liver and lungs are the most common sites, with skeletal involvement being rare [3,4]. Compared with clear-cell RCC, the most common and aggressive subtype, ChRCC is distinguished by its lower metastatic rate, pale eosinophilic cytoplasm, and characteristic perinuclear halos on histology.

Conversely, multiple myeloma (MM) is a plasma cell malignancy that frequently presents with osteolytic bone lesions, which represent areas of bone resorption caused by tumor-mediated osteoclast activation, mimicking metastatic skeletal disease [5]. MM is characterized by clonal proliferation of plasma cells in the bone marrow, leading to bone destruction, hypercalcemia, anemia, and renal dysfunction. The spine is a common site of involvement, and patients often present with persistent back pain and vertebral fractures [6]. In the context of a patient with ChRCC, the development of new back pain and lytic bone lesions typically raises suspicion for metastatic disease. However, distinguishing between metastatic RCC and a new diagnosis of MM is critical, as the management strategies and prognoses differ significantly.

Cases of synchronous or metachronous occurrence of RCC and MM have been reported, though they are exceedingly rare [7]. The pathophysiological association between these two malignancies remains unclear, but their coexistence underscores the importance of maintaining a broad differential diagnosis in patients with a history of cancer who present with new skeletal symptoms [8,9]. The overlap in clinical presentation between metastatic RCC and MM creates a significant diagnostic challenge. Both conditions can present with similar radiologic findings, namely, lytic bone lesions, necessitating a thorough diagnostic workup. This includes advanced imaging, serum protein electrophoresis, and often a bone marrow biopsy to establish a definitive diagnosis. Misdiagnosis can lead to inappropriate treatment and adversely affect patient outcomes. Here, we report a rare case of MM occurring in a patient with pre-existing ChRCC, emphasizing the diagnostic challenges and the importance of differentiating between metastatic disease and a second primary malignancy.

## Case presentation

**Figure 1:**
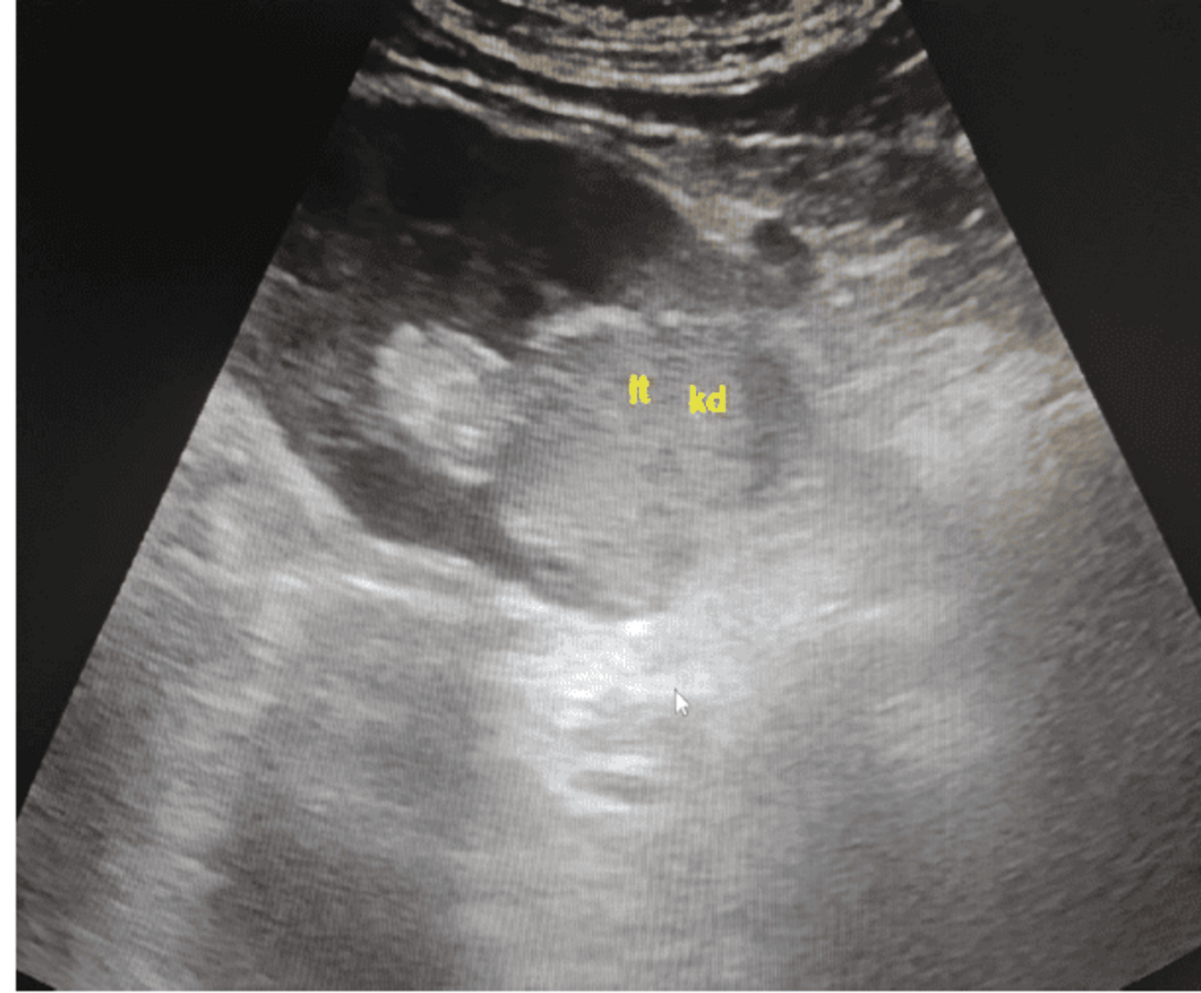
Ultrasound image of the left kidney demonstrating a heterogeneous hypoechoic mass involving approximately the lower one-third of the renal parenchyma. This finding raised suspicion of a renal tumor and prompted further imaging.

A 66-year-old male presented to the urology clinic with persistent back pain. An ultrasound performed outside the hospital identified a 3 mm lower calyceal stone in the left kidney and raised suspicion of a lower-pole angiomyolipoma. A follow-up ultrasound conducted at Bahrain Defence Hospital confirmed the presence of a heterogeneous hypoechoic mass involving approximately the lower one-third of the left renal parenchyma, while the right kidney appeared normal (Figure 1).

**Figure 2:**
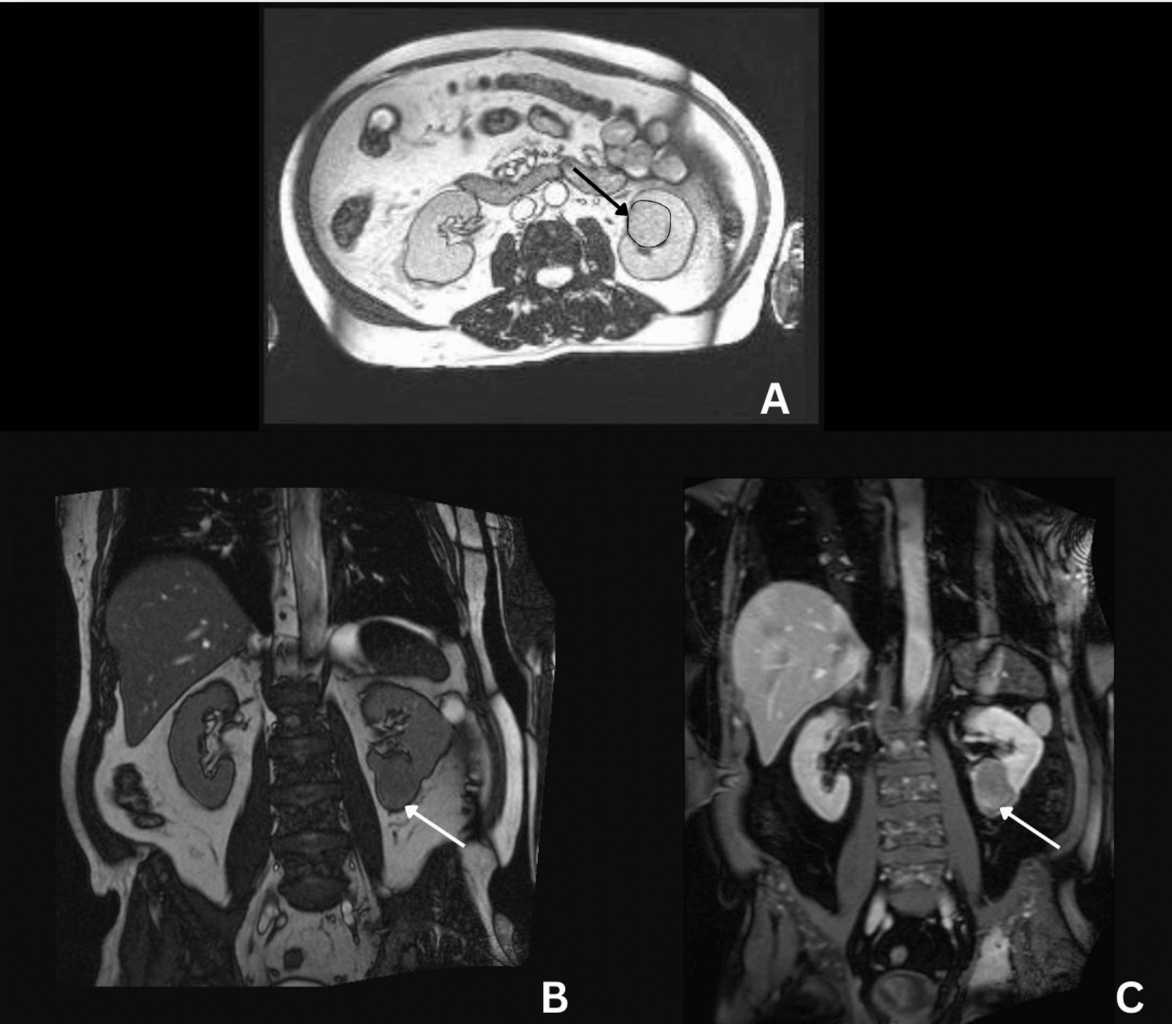
(A) Transverse T2-weighted abdominal and pelvic MRI. (B) Coronal T2-weighted abdominal and pelvic MRI. (C) Coronal T1-weighted post-contrast abdominal and pelvic MRI. All images reveal a suspicious left renal mass with multiple vertebral lytic lesions, suggestive of metastatic disease.

Because of mild renal impairment (estimated glomerular filtration rate of 56 mL/minute/1.73 m², Modification of Diet in Renal Disease formula), a contrast-enhanced CT was deferred, and instead an MRI of the abdomen with contrast (non-dedicated renal protocol, no dynamic contrast-enhanced phases) was performed. The MRI demonstrated a 55 × 55 × 35 mm lobulated mass in the lower pole of the left kidney, showing low signal intensity on both T1- and T2-weighted sequences, slight restricted diffusion, no intralesional fat, and no appreciable post-contrast enhancement. The lesion extended into the perirenal space but without invasion beyond Gerota’s fascia, consistent with at least T3a staging. No regional lymphadenopathy was detected (N0). In addition, multiple lytic bony lesions were noted, raising suspicion of skeletal metastases (Figure 2).

**Figure 3:**
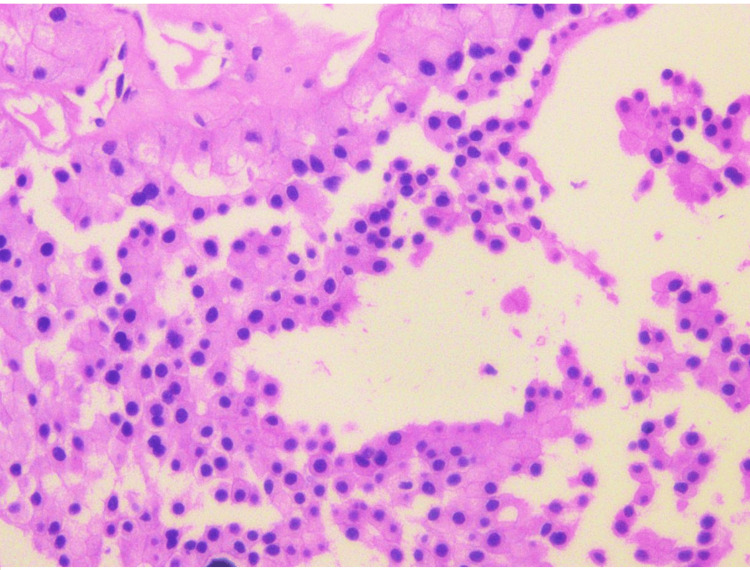
High-power magnification showing chromophobe renal cell carcinoma (hematoxylin and eosin, 400×).

**Figure 4:**
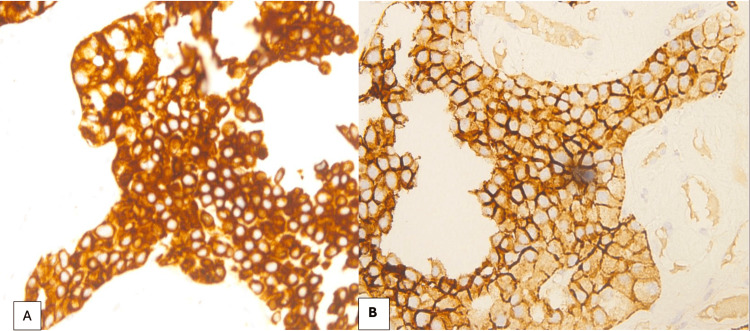
Immunohistochemical staining of the renal cell carcinoma showing strong and diffuse positivity for CK7 (A) and C-Kit (B).

Based on the initial MRI findings, which demonstrated multiple bony lesions, the urology team, guided by European Society for Medical Oncology recommendations, considered systemic/palliative therapy for presumed metastatic RCC, rather than a curative surgical approach, as the extent of the disease suggested advanced, non-curable status. An ultrasound-guided biopsy of the renal mass was subsequently obtained, which revealed ChRCC (Figures 3, 4), a histological subtype known for its relatively indolent behavior and low metastatic potential. Given this unexpected finding, the case was presented at the multidisciplinary tumor board (MDT). The board members noted that the extensive skeletal involvement appeared disproportionate to the relatively small size of the primary tumor (55 mm) and the ChRCC subtype, raising concern that the skeletal lesions may not, in fact, represent metastatic disease. This prompted further investigation.

**Figure 5:**
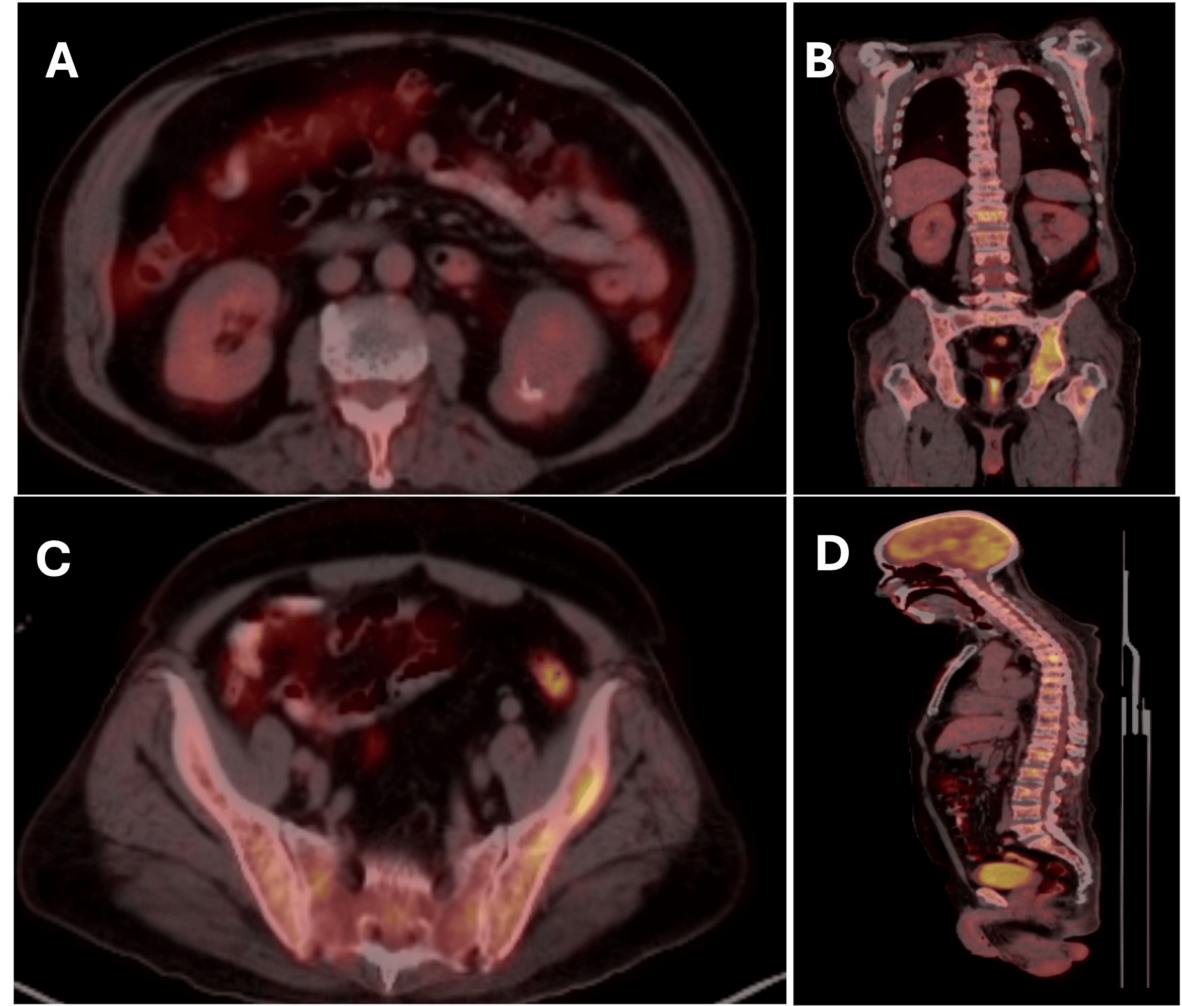
FDG-PET/CT scan displaying low-grade metabolic activity of the renal mass and widespread hypermetabolic lytic skeletal lesions. (A) Fused axial images show low-grade metabolic activity associated with the left lower pole renal mass containing calcifications (SUVmax ≈ 2.7). (B) Fused coronal images reveal extensive hypermetabolic lytic osseous lesions involving multiple vertebral bodies and the pelvis. (C) Fused axial images demonstrate multiple hypermetabolic lytic lesions within the pelvic bones (SUVmax ≈ 8.4). (D) Fused sagittal images show diffuse hypermetabolic lytic osseous lesions affecting the vertebral bodies and posterior elements of the thoracic and lumbar spine (SUVmax ≈ 7). FDG-PET/CT: fluorodeoxyglucose-positron emission tomography/computed tomography; SUVmax: maximum standardized uptake value

**Figure 6:**
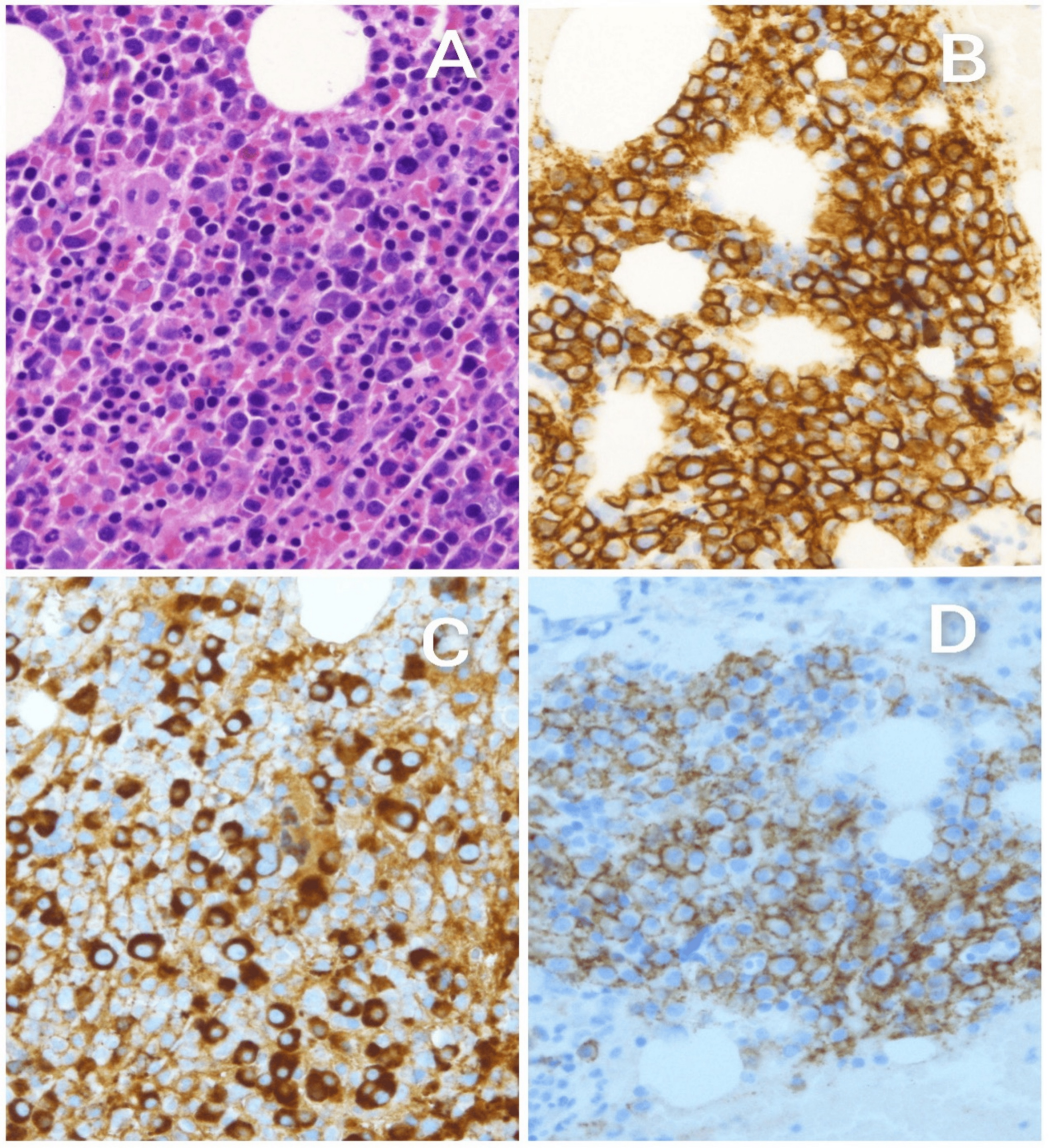
(A) High-power view showing hypercellular bone marrow with infiltration by sheets of plasma cells (hematoxylin and eosin, 40×). (B-D) Immunohistochemical studies: (B) CD138 highlights plasm cells. (C) Kappa light chain restriction: CD56 positivity.

A positron emission tomography (PET)/CT scan was then performed on January 31, 2024, which showed low-grade metabolic activity in the renal mass but revealed widespread hypermetabolic lytic lesions involving the axial and appendicular skeleton (Figure 5). A small lung nodule was also noted, but was of uncertain significance. A bone biopsy was pursued and ultimately confirmed the diagnosis of MM (IgG kappa type) (Figure 6). Laboratory results were consistent with the diagnosis (Table 1).

**Table 1:** Laboratory findings of the patient.

Test	May 2024	May 2025 (post-treatment)	Reference range
IgG	6,302.7 mg/dL	714.6 mg/dL	681–1,648 mg/dL
Kappa/Lambda ratio	1.19	2.28	0.31–1.56
Immunofixation	IgG kappa monoclonal band	IgG kappa monoclonal band	Negative
Beta-2 microglobulin	3.87 g/L	2.82 g/L	2.3–4.7 g/L

Bone marrow biopsy demonstrated 60% plasma cell infiltration, consistent with the International Staging System Stage III developed by the International Myeloma Working Group (IMWG). The patient was started on a DVD regimen (daratumumab, bortezomib, dexamethasone) along with zoledronic acid. After four cycles, there was an 80% reduction in M-protein, but bone marrow biopsy still showed 20-50% plasma cells, so lenalidomide was added. Following further therapy, the patient underwent autologous stem cell transplantation with high-dose melphalan conditioning. Post-transplant, the patient achieved a very good partial response and minimal residual disease-negative status and was started on lenalidomide and zoledronic acid maintenance therapy.

Regarding the renal lesion, management was reviewed in a joint Urology-Radiology meeting, where surgical intervention was considered the most appropriate approach. Radical nephrectomy was favored over partial nephrectomy due to the tumor’s size and proximity to the renal hilum, while non-surgical ablation options were deemed unsuitable. The case was subsequently scheduled for formal discussion at the Tumor Board MDT to confirm the adequacy of ongoing MM treatment and finalize the RCC management plan.

At the latest follow-up, 2.5 months post-transplant, the patient was clinically well with unremarkable laboratory results. Continued monitoring and supportive care were emphasized to ensure the best possible outcomes for the patient’s overall health.

A contrast-enhanced CT scan of the abdomen performed in May 2025 demonstrated no interval change in the left renal mass compared with the MRI of the abdomen from January 2024, indicating radiological stability. The CT also re-demonstrated multiple lytic bony lesions.

## Discussion

The coexistence of RCC and MM is exceptionally rare but increasingly recognized in the literature. There has been a good number of cases reported of RCC occurring alongside MM; however, the vast majority of these cases involve the clear cell subtype of RCC. ChRCC, in particular, has virtually never been described in association with MM. The relative risk of MM incidence is 51% higher among patients with RCC than in the general population, and the relative risk of RCC incidence is 89% higher among MM patients [10]. ChRCC, in particular, is characterized by an indolent course and a low rate of bone metastasis, with skeletal involvement reported in fewer than 7% of cases [1]. In contrast, MM frequently presents with diffuse osteolytic bone lesions and back pain, features that can closely mimic metastatic bone disease from RCC [5].

This clinical overlap presents a significant diagnostic challenge, as the management of bone metastases from RCC differs markedly from that of MM bone disease. RCC bone metastases are typically managed with surgery, radiotherapy, and targeted therapies such as tyrosine kinase inhibitors and immune checkpoint inhibitors, while MM requires systemic chemotherapy, immunomodulatory agents, and bisphosphonates to address bone involvement [11,12]. Therefore, accurate differentiation is crucial before initiating therapy.

Several diagnostic tools assist in distinguishing between these entities. MM is characterized by clonal plasma cell proliferation in the bone marrow, monoclonal protein (M-protein) in serum or urine, and end-organ damage (CRAB criteria: hypercalcemia, renal dysfunction, anemia, bone lesions), as defined by the IMWG (2015) [13]. Imaging in MM often reveals multiple, well-circumscribed lytic lesions without sclerotic borders, whereas RCC bone metastases may appear as solitary or fewer lesions, sometimes with associated soft tissue masses [14]. Laboratory markers such as elevated beta-2 microglobulin, abnormal serum free light chain ratios, and high immunoglobulin levels are more indicative of MM. Bone marrow biopsy and advanced imaging modalities (PET/CT, MRI) are essential for definitive diagnosis [13].

The pathophysiological connection between RCC and MM remains under investigation. Both malignancies may exploit similar cytokine environments, particularly interleukin-6, which supports tumor proliferation and survival [9]. Shared risk factors, such as obesity, hypertension, and smoking, may also contribute to their co-occurrence [15]. The coexistence of RCC and MM within the same bone lesions may be partly explained by their reliance on common oncogenic signaling pathways, particularly the PI3K/AKT/mTOR pathway, which regulates cell growth, survival, metabolism, and disease progression in both malignancies [16,17]. This could suggest that tumor removal could disrupt these shared pathways, potentially reducing myeloma burden [18]. This is consistent with pathological findings of intermingled tumor cells and the known biology of tumor-microenvironment interaction [19].

## Conclusions

This case highlights the diagnostic challenge of distinguishing between RCC metastasis and MM in patients presenting with new bone pain and lytic lesions. Clinicians should be aware of the potential for dual malignancies and ensure thorough evaluation to avoid misdiagnosis. Multidisciplinary collaboration and comprehensive diagnostic protocols are essential for optimal management and improved patient outcomes in such complex presentations. Future research should continue to explore the interplay between different malignancies, which may further refine diagnostic and treatment strategies, and the effect of nephrectomy on MM outcomes.
